# CircRASSF2 acts as a prognostic factor and promotes breast cancer progression by modulating miR-1205/HOXA1 axis

**DOI:** 10.1080/21655979.2021.1933300

**Published:** 2021-06-28

**Authors:** Wei Zhong, Lei Bao, Yangyi Yuan, Yanzhi Meng

**Affiliations:** a Department of Breast Cancer, Hubei Cancer Hospital, Tongji Medical College, Huazhong University of Science and Technology, Wuhan, Hubei, China; b Department of Pathology, The Affliated Yantai Yuhuangding Hospital of Qingdao University, Yantai, Shandong Province, China; c Fuzhou Medical College, Nanchang University, Nanchang, Jiangxi Province; d Department of Medical Ultrasonics, Wuhan Youfu Hospital, Wuhan, Hubei, China

**Keywords:** Breast cancer, circRASSF2, miR-1205, proliferation, migration, invasion

## Abstract

Circular RNA (circRNA), a recently identified endogenous non-coding RNA molecule, regulates gene expression in mammals. At the current stage, the expression and function of circRASSF2 in breast cancer (BC) have not been clarified. According to our study, it is found that circRASSF2 sequences contain miR-1205 binding sites, and Homeobox gene A1 (HOXA1) is the target gene of miR-1205. Besides, the clinical observations and histopathologic study reveal that the expression of circRASSF2 increased to a significant extent in BC tissues and serum. Additionally, it is found that circRASSF2 expression had a positive correlation with distant metastasis, lymph node metastasis, TNM stage, differentiation and tumor size, and that overall survival (OS) and progression-free survival (PFS) of circRASSF2 high expression BC patients were inferior to those with low circRASSF2 expression. In vitro study, an overt decrease was detected in the proliferation, clone formation ability, migration and invasion of breast cancer cells in cells when circRASSF2 was knocked down. We confirmed the direct interaction between circRASSF2, miR-1205 and HOXA1 by a dual luciferase reporter system. Additionally, our study revealed that over-expression of miR-1205 decreased HOXA1 protein expression, and HOXA1 protein expression decreased when circRASSF2 were knocked down, and when miR-1205 expression was inhibited, HOXA1 expression was significantly increased. In conclusion, our study suggests that circRASSF2 regulates BC progression through the miR-1205/HOXA1 pathway. Our findings suggest the prospect of circRASSF2 serving as therapeutic target as such to cure BC patients.

## Introduction

Breast cancer (BC) is the most ubiquitous cancer threatening female health globally. BC takes up 30% of cancer in women, and the incidence and mortality of BC may continue to rise as global life expectancy increases [1]. Treatment options for BC include surgery, chemotherapy, radiotherapy and biologically targeted therapy. Surgery to remove tumor breast tissue remains the most commonly method [2]. Some patients have already developed distant metastasis when they are diagnosed, resulting in a poor prognosis. Therefore, the investigation of the pathogenesis of BC and screening for specific biological markers for treatment and diagnosis is a current research priority.

CircRNAs, a kind of endogenous non-coding RNA molecules detected recently [3]. CircRNAs differ structurally from normal linear RNA molecules in that they do not have 5ʹ and 3ʹ ends or a polyadenylated tail. CircRNA exists in the body as a closed ring structure and cannot be degraded by RNA enzymes. The cytoplasm of eukaryotic cells exhibits a high level of circRNA expression. Notably, the expression is stable, abundant and conserved, usually expressed in specific tissues or specific developmental stages, and is characterized by strong tissue and cell specificity [4]. As is shown by relevant studies, the biological function of circRNA is similar to that of endogenous competitive RNA (ceRNA), ceRNA is accomplished by tiny RNA response elements (MRE). CeRNA has different numbers and kinds of MRE that are able to prevent micro-RNA (miRNA) from refraining its target gene when combined with miRNA. There are several complementary binding sites of miRNA on circRNA, which can be used as the ‘sponge’ of RNA to absorb miRNA, so as to relieve downstream target genes from being negatively regulated by miRNA [5–7].

CircRASSF2 (hsa_circ_0059354) is highly expressed in laryngeal and thyroid carcinomas and plays the role of an oncogene [8], but there are no reports on the expression and role of circRASSF2 in BC. As a small class of non-coding RNA, miRNA is responsible for gene expression during post-transcription stage through targeted mRNA, leading to degradation or translation inhibition of target mRNA [9]. The up-regulation of carcinogenic miRNA will lead to the down-regulation of tumor suppressor gene expression, on the contrary, the down-regulation of tumor suppressor miRNA will lead to the increase in oncogene expression [10]. In tumor research, circular RNA has been widely reported as the mechanism of downstream target genes regulated by the ‘sponges’ of miRNA [11,12], and has great potential in diagnosing, treating and prognosticating breast, liver and bladder cancer [13–15]. MiR-1205 is reported to exhibit a low level of expression in many types of tumors, and its expression is relative to tumorigenesis, progression, and prognosis of tumors [16,17]. However, the function of miR-1205 in BC remains to be explored.

Several findings can be drawn based on our previous study. To begin with, circRASSF2 sequence contains miR-1205 binding site. In the second place, Homeobox gene A1 (HOXA1) was the target gene of miR-1205, and was highly expressed in a variety of tumors [18,19]. HOXA1 belongs to the Hox gene family and comprises the cluster on chromosome 7 to coordinate the development of various cell behavior patterns during embryogenesis [20], and abnormal expression of HOXA1 in differentiated cells may lead to the acquisition of tumor-promoting properties [21].

The present study is intended to explore the expression, function and molecular mechanism of circRASSF2 in BC, which is exploited as a miR-1205 sponge to adjust how HOXA1 is expressed and thus promote BC cells proliferation, migration and invasion via in vitro study. It is expected that our research will lead to a deeper understanding of BC and ascertain a novel effective therapeutic target for BC.

## Materials and methods

### Patients and samples

Tissue samples: The paired samples of 70 BC along with matched noncancerous normal tissues came from BC patients who were operated on between 2013 and 2019, with a median age of 48. Clinicopathological characteristics in our study included tumor size, degree of differentiation and TNM staging, distant metastasis and lymph node metastasis. Notably, none of the patients was previously treated with chemotherapy or radiotherapy before surgery. Immediately after removal of the specimens, the tissue was plunged into liquid nitrogen for preservation.

Serum samples: new cases of BC were collected between 2013 and 2019, excluding those with other types of cancer and not treated with radiotherapy or chemotherapy prior to diagnosis.

With the approval from the Ethics Committee at the Wuhan Youfu Hospital, all the specimens in this study were acquired as is stipulated by the ethical guidelines of the 2013 Declaration of Helsinki, and we have obtained informed consent from all the patients involved.

### Cell culture

Human BC cell lines (T47D, MCF-7, BT549, Hs-578 T, MDA-MB-231 and MDA-MB-468) were purchased from Cell Bank of Chinese Academy of Sciences (shanghai, China). RPMI 1640 medium that consist of 10% Fetal bovine serum (FBS, Invitrogen) and 1% Penicillin-Streptomycin was used to maintain T47D cells. DMEM media containing 10% FBS, and 1% penicillin/streptomycin was used for maintaining MCF-7 cells. BT-549 cells were preserved in RPMI 1640 with 10% FBS. MDA-MB-231, Hs578T and MDA-MB-468 cells were maintained in DMEM with 10% FBS and 1% Penicillin-Streptomycin. MCF-10A cells were maintained in DMEM/F12 (5% horse serum, 100 ng/ml cholera toxin, 10 μg/ml insulin, 500 ng/ml hydrocortisone, 20 ng/ml epidermal growth factor) [22]. As for the culture of the cells, a cell incubator at 37°C and 5% CO_2_ was required. The experimental cells were all in the logarithmic growth phase. Cell culture medium, penicillin-Streptomycin, FBS, horse serum, cholera toxin and other factors were all purchased from Life Technologies (Gibco, USA).

### Lenti virus infection and cell screening

Cells were transplanted to 6-well plates whose density is 1 × 10^6^ cells/mL for 12 h growth at 37°C and 5% CO_2_, and viral infections should be performed when the cell density reached 70%-80% confluence. Recombinant lentiviruses si-circRASSF2 (LV3-si-circRASSF2#1, LV3-si-circRASSF2#2, LV3-si-circRASSF2#3), si-NC (LV3-NC)) were used to transduce cells; the infected cells were placed in a cell culture incubator for 24 h at 37°C and 5% CO_2_, the culture medium containing virus and polybrene were removed, added fresh complete medium per well and continued to incubate for 48 h at 37°C and 5% CO_2_, the efficacy of infection was detected by qRT-PCR. That is to say, suppose the infection rate was greater than 60%, puromycin (2 μg/ml) was added to the cells for 48 h to further selection of infected cells. In subsequent experiments, the stable knockdown of low circRASSF2-expressing cell lines as well as control cells were put to use. The lentiviral vector used in this study was pHBLV-CMV-crRNA-EF1-GFP-T2A-puro (HanBioCo., Ltd., Shanghai, China). si-RNA was synthesized by Guangzhou Ribo BioCo. The RNA sequcence was listed below: si-circRASSF2#1:AAGAGAGTGGCCCAGTATATA,si-circRASSF2#2:AGAGAGTGGCCCAGTATATAA,si-circRASSF2#3:AAAGAGAGTGGCCCAGTATAT.

### Cell transfection

With the assistance of Lipofectamine 3000 (Invitrogen, CA, USA), transfection experiments were implemented. First, trypsin was used to digest cells which were then resuspended, and inoculated into new well plates whose density is 3 × 10^5^ cells/well and cultured for 12 h. When the confluence of cells reached 70–80%, according to manual instruction, miR-1205 mimic, miR-1205 control or miR-1205 inhibitor (GenePharam, Shanghai, China) was transfected. The sequence was listed below: hsa-miR-1205 mimic: 5ʹ-UCUGCAGGGUUUGCUUUGAG (sense), 5‘-CUCAAAGCAAACCCUGCAGA-3′ (anti-sense); has-miR-1205 inhibitor,CUCAAAGCAAACCCUGCAGA; scramble control: 5′-UUCUCCGAACGUGUCACGUTT-3′

### CCK-8 assay

After the transfection, 96-well plates with a density of 2.5 × 10^3^/well were plated with the cells. Thereinto, each well had a total volume of 200 μl medium and then incubated at 37°C and 95% CO_2_. After 12 h, the medium was removed, and 10 µl (5 mg/mL) of CCK-8 (Tokyo, Japan) and 190 µl of complete medium were added and placed in a cell culture incubator for 2 h. A multifunctional enzyme spectrometer was employed to measure the 0 h, 24 h, 48 h, 72 h OD value at 450 nm.

### Clone formation assay

With the help of 0.25% trypsin, the cells were digested so as to prepare single-cell suspensions. Six well plates were seeded with 4 × 10^5^ cells/well and gently rotated to evenly disperse the cells, and the cells were continuously cultured until visible clones appeared in the petri dish. After being fixed for 15 minutes in 5 mL with the addition of 4% paraformaldehyde, cells were removed from the fixative, 2 ml GIMSA staining solution was added for 15 minutes, and the cells were gently rinsed three times with PBS. After slow washing of the staining solution with running water, the stained plate was placed under an inverted microscope to count cell colonies of more than 10 cells. Cell colony formation rate represents the ratio of the number of cell colonies to that of inoculated cells in each group, and the cell radial survival fraction was calculated from the colony formation rate, clone formation rate = (number of clones/number of inoculated cells) × 100%.

### Flow cytometry detection

Upon the preparation of single-cell suspension, cells were centrifuged to remove the supernatant at 1000 g for 10 mins at 4°C, the density of cells was tuned to 5 × 10^5^ cells/mL. Then, pre-cooled PBS was used to wash the cells. After 10-min centrifugation at 1000 g, 4°C, cell culture supernatant was filtered out. When the cells were harvested, Annexin V-PE and 7-Aminoactinomycin D (7AAD) was exploited to stain these cells as is prescribed by the manufacturer’s instruction (BD Biosciences, CA, USA). Cells were incubated with 5 μL of PE-labeled AnnxinV and 5 μL of 7AAD for 15 minutes at room temperature without light. A flow cytometry (BD Biosciences, Franklin Lakes, NJ, USA) was used for the analysis of cell apoptosis.

### Dual luciferase assay

With the help of Dual-Luciferase System, luciferase assay was performed (Promega, WI, USA). The plasmid wild or mutant circRASSF2/HOXA1 and miR-1205 mimic or negative control (miR-NC) were cotransfected into the cells with Lipofectamine 3000 (Invitrogen, CA, USA). The experimental groups were as follows: PGL3-circRASSF2 WT+ miR-1205 mimic, PGL3-circRASSF2 WT+miR-NC, PGL3-circRASSF2 MUT+miR-1205 mimic, PGL3-circRASSF2 MUT + miR-NC; pGL-3-HOXA1 WT+ miR-1205, pGL-3-HOXA1 WT+ miR-NC, pGL-3-HOXA1 MUT+ miR-1205, pGL-3-HOXA1 MUT+ miR-NC. After 48 hours, the luciferase activity was found to be normalized to Renilla luciferase activity.

### qRT-PCR assay

Different groups of transfection cells MCF7 and BT549 (si-NC and si-circRASSF2) were taken from the logarithmic growth phase, and the cell density was 5 × 10^6^ cells/mL. The extraction of total cell RNA was fulfilled by the TRIzol method (Invitrogen, Carlsbad, CA), and a reverse transcription kit (Invitrogen) was used for cDNA synthesis. By virtue of the SYBR Green PCR Master Mixing Kit (ABI, Applied Biosystems), qRT-PCR was performed. 18rRNA and U6 internally regulate circRASSF2 and miR-1205, respectively. 2-∆∆CT method was applicable to the measurement of relative expression levels. The qPCR primer sequences are listed as below: circRASSF2; F: 5ʹ-CTTTTCAAAGAGAGTGGCCCAG,R:5ʹ-ACGGTGTACTTGCGCATCAG-3ʹ; has-miR-1205, F: 5ʹ-GCAGGGTTTGCTTTGAGTACTTCCTTCCTGTCA-3ʹ, R-5ʹ-GTCCAGTTTTTTTTTTTTTTTACAFACT-3ʹ; 18SrRNA, 5ʹ-CGGCTACCACATCCAAGGAA-3ʹ, R: 5ʹ-GCTGGAATTACCGCGGCT-3ʹ; U6, F: 5ʹ-CGCTTCGGCAGCACATAT-3ʹ, R: 5ʹ-AAATATGGAACGCTTCACGA-3ʹ.

### Western blot assay

The cell density was 5 × 10^6^ cells/ml. The extraction of total cell protein was fulfilled by RiPA lysis (Bio-technology, Shanghai, China), and protein concentration was monitored by BCA protein quantification kit (Bio-technology, Shanghai, China). Each well on a 12% SDS-PAGE gel was loaded with 50 μg of proteins via electrophoresis at 100 V for 100 min, and subsequently transferring proteins to PVDF membrane was completed (Millipore, MA, USA) at 200 V for 60 mins. Upon the completion of the transfer, the PVDF membrane was locked down for 1 h in 5% skimmed milk at room temperature. Afterward, the membrane was rinsed with TBST three times with each time lasting for 10 minutes; after the incubation of the membrane in primary antibodies PCNA (1:500, Abcam, Cambridge, MA), PARP (1:500, Abcam), Cleaved-PARP (1:1000, Abcam), caspase 3 (1:1000, Abcam), cleaved-caspase 3 (1:1000, CST), MMP2 (1:500, Abcam), MMP9 (1:200, Abcam) at 4°C overnight, it was then rinsed three times for 5 min each with TBST; it was an indispensable procedure to incubate the membrane in secondary antibodies conjugated with horseradish peroxidase (1:3000, Abcam) for 1 h at room temperature. The membrane was rinsed with TBST again. Finally, the Western Bright ECL kit (Advansta, CA, USA) was adopted to make the protein band visible, while the quantification of relative protein expression was achieved by ImageJ software.

### Transwell migration and invasion assay

Different groups of transfection cells MCF7 as well as BT549 (si-NC and si-circRASSF2) were taken from the logarithmic growth phase, and after being digested with trypsin, the cells were resuspended in serum-free medium with 3 × 10^4^ cells/ml. Transwell chambers were coated with 5 mg of Matrigel (BD, CA, USA) diluted with serum-free medium mixture with a volume of 20 μl and allowed to air dry overnight as for invasion assay. 200 μl of medium containing cells and 500 μl of medium containing 20% FBS were added to the upper chamber and to the bottom chamber, respectively. After 24-h incubation, cotton swabs were used to remove the upper chamber nonmigratory cells, and the remaining cells were fixed by 4% paraformaldehyde for 30 mins, and then stained with 0.1% crystalline violet for 30 mins. The membranes were washed with PBS, and finally the cells were photographed and counted under an orthomolecular microscope at 200× magnification.

### Statistical analysis

In this part, SPSS 22.0 software (IBM Corp, Armonk, NY, USA) was adopted to carry out statistical analysis of experimental data. The experiment was conducted more than three times. Data were expressed as x ± s. The comparison between two groups was conducted by LSD-t test, and that between groups was implemented via independent samples t-test. One-way ANOVA was used for comparing multiple groups, and the correlation of clinicopathological data was analyzed via the chi-square test. P < 0.05 in chi-square test indicates a statistically significant difference.

## Results

This study strove to investigate circRASSF2 mechanism in BC development. A high level of circRASSF2 expression was observed in BC tissues and cells, which was bound up with the overall survival of BC patients negatively. In vitro studies, CCK8 assay, cell clone formation assay and Transwell assay indicated that circRASSF2 knockdown hampered cells from proliferating, invading and migrating. circRASSF2 knockdown enhanced miR1205 expression, but refrained HOXA1 expression. What is more, it is highly possible that HOXA1 inhibits cell proliferation, invasion and migration, whilst the effect of circRASSF2 knockdown was relieved by the increase in HOXA1 expression. It is thus speculated that circRASSF2 conduces to cell proliferation, invasion and migration in BC by refraining miR1205 and accelerating HOXA1 expression.

### CircRASSF2 is upregulated in BC tissue and correlates with the progression and poor prognosis

**Figure 1.**
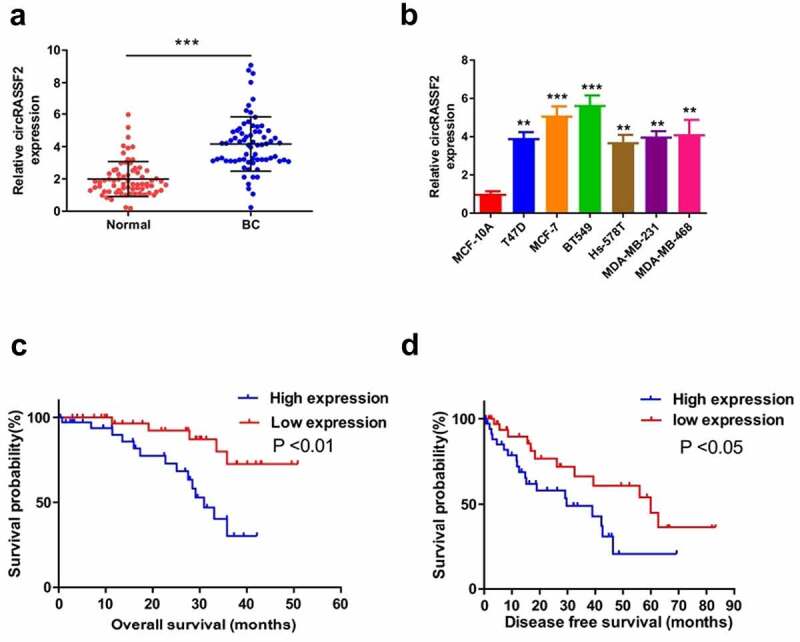
**CircRASSF2 is upregulated in BC tissue and correlates with the progression and poor prognosis** (a) The expression levels of circRASSF2 in BC tissue and normal tissue were detected by qRT-PCR. (b) The expression levels of circRASSF2 in BC cell lines (T47D, MCFMFC, MCFMU7, BT549, Hsmur578, MDAMUMU231, MDAMurMBMUL468) and Normal breast epithelial MCF-10A cells were detected by qRT-PCR. (c) Kaplan-Meier survival curve was used to evaluate the overall survival time of BC patients with low expression of circRASSF2 (n = 35) and high expression of circRASSF2 (n = 35). (d) Kaplan-Meier survival curve was used to evaluate the progression-free survival time of BC patients with low expression of circRASSF2 (n = 35) and high expression of circRASSF2 (n = 35). *, P < 0.05; **, < 0.01; ***, P < 0.001

The expression levels of circRASSF2 were observed via qRT-PCR. As demonstrated by the results, circRASSF2 expression was up-regulated to a significant extent in BC tissues in comparison with noncancerous normal tissues (Figure 1(a), p < 0.001). The expression of circRASSF2 in six BC cell lines (T47D, MCF-7, BT549, Hs-578 T and MDA-MB-231, MDA-MB-468) were significantly up-regulated compared to normal breast epithelial MCF-10A cells (Figure 1(b), p < 0.001).

Using the median expression value of circRASSF2 as cutoff value, 70 BC patients were divided into two groups, namely low expression (n = 35, expression values ≤ median value) and high expression (n = 35, expression values > median value). By virtue of the chi-square test, the relationship between circRASSF2 expression and clinicopathological data was calculated. The results showed that circRASSF2 expression had positive correlation with distant metastasis, lymph node metastasis, TNM stage, differentiation and tumor size (Table 1, p < 0.001), but was independent of patient age and gender (Table 1, P > 0.05). In addition, the OS and PFS time of BC patients in the circRASSF2 high expression group were obviously shorter than those in the low expression group (Figure 1(c,d), P < 0.001). The results suggest that circRASSF2 is closely related to BC development and prognosis, and can be used as a feasible biomarker in the course of BC prognosis treatment.

**Table 1.** Correlations of CircRASSF2 expression with clinicopathologic features of breast cancer patients

Parameters	Total	circRASSF2		P-value
		LOW(35) High(35)		
**Age**				0.488
>50	42	22	20	
≤50	28	13	15	
**Tumor size** >2 cm	41	16	25	0.029
≤ 2 cm	29	19	10	
**differentiation**				0.016
High (well)	32	11	21	
Low (poor)	38	24	14	
**TNM stage**				0.015
I–II	28	19	9	
III – Ⅳ	42	16	26	
**Lymph node metastasis**				0.031
Negative	37	23	14	
Positive	33	12	21	
**distant metastasis**				0.03
M0	31	20	11	
M1	39	15	24	
**ER status**				0.089
Positive	29	18	11	
Negative	41	17	24	
**PR status**				0.203
Positive	23	9	14	
Negative	47	26	21	
**HER-2 status**				0.029
Positive	29	10	19	
Negative	41	25	16	

### CircRASSF2 exbibits a high level of expression serum of patients with BC and can be a good diagnosis marker

**Figure 2.**
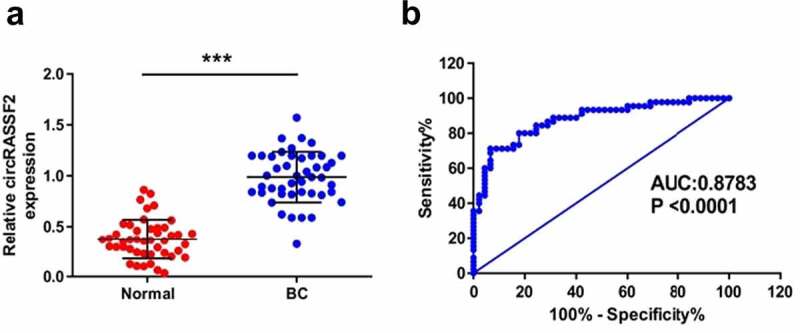
**CircRASSF2 is highly expressed in serum of patients with BC and can be a good diagnosis marker. (**a) The expression levels of circRASSF2 in BC serum were detected by qRT-PCR. (b) ROC analysis was used for calculating predictive values. *, P < 0.05; **, < 0.01; ***, P < 0.001

For the sake of discerning the expression level of circRASSF2 in serum of 45 BC and 45 normal subjects, qRT-PCR was carried. As the results implied, there was a clear increase in the expression of circRASSF2 in the serum of BC patients relative to normal subjects (Figure 2(a), p < 0.001).

The serum circRASSF2 expression level was further analyzed using receiver operating characteristic (ROC) curves to predict the presence of BC. The results showed that the area under the ROC curve (AUC) was 0.9323, suggesting that circRASSF2 is a good biomarker for BC diagnosis.

### Knockdown of circRASSF2 inhibits BC cells proliferation and induces apoptosis

**Figure 3.**
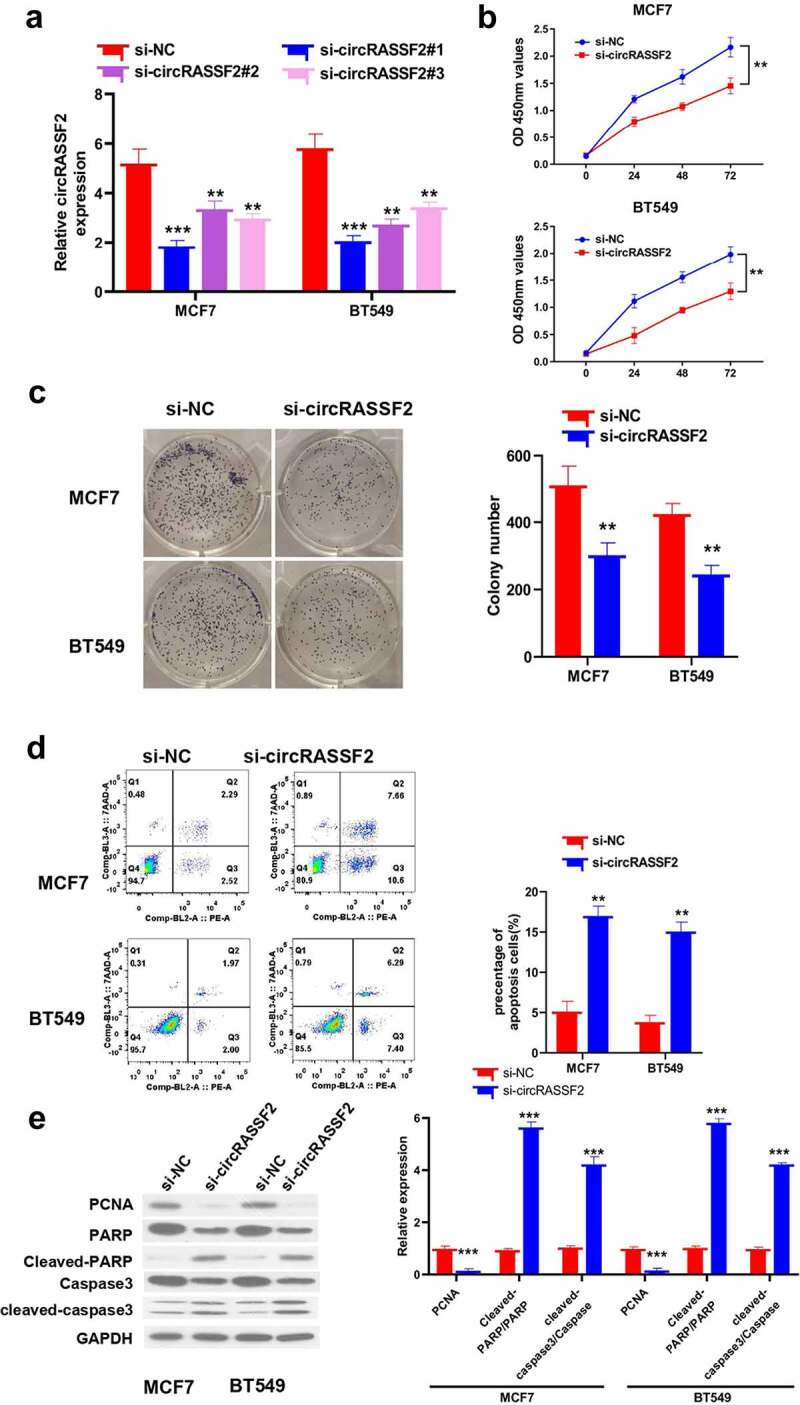
**Knockdown of circRASSF2 inhibits BC cells proliferation and induced apoptosis**. (a) The knocking efficiency was detected by qRT-PCR. (b) CCK-8 assay was used to detect the proliferative activity in different groups of MCF7 and BT549 (si-NC and si-circRASSF2) at different times (0 h, 24 h, 48 h and 72 h). (c) Clone formation assay was used to detect the clone formation ability of different groups of MCF7 and BT549 cells (si-NC and si-circRASSF2). (d) Flow cytometry was used to detect the level of apoptosis in different groups of MCF7 and BT549 (si-NC and si-circRASSF2). (e) Western blotting assay was used to detect the protein levels of PCNA, Cleaved-PARP and cleaved-caspase3 in different groups of MCF7 and BT549 (si-NC and si-circRASSF2). *, P < 0.05; **, < 0.01; ***, P < 0.001

MCF7 and BT549, which have relatively high expression in circRASSF2 in BC cell lines, were selected for knockdown of circRASSF2 by lentiviral infection transfection, and three sequences of circRASSF2 siRNA (si-circRASSF2#1, si-circRASSF2#2, and si-circRASSF2#3) were used, and the expression levels of circRASSF2 in MCF7 and BT549 cells transfected with circRASSF2 were significantly lower than those transfected with si-NC (Figure 3(a), p < 0.01), and the knock-down with si-circRASSF2#1 had the best effect was chosen for subsequent experiments.

The proliferation of MCF7 and BT549 cells was investigated by CCK-8 assay in different groups (si-NC and si-circRASSF2) at different times (0 h, 24 h, 48 h and 72 h). It was implied that the proliferative activity of cells in si-circRASSF2 group was hampered greatly relative to that of si-NC group cells, and the difference was most obvious at 72 h (Figure 3(b), p < 0.01). The clonogenic ability of MCF7 and BT549 cells in different groups (si-NC and si-circRASSF2) were examined. As is exhibited by the results, si-circRASSF2 knockdown inhibited the clonogenic ability of cells (Figure 3(c), p < 0.01), while circRASSF2 knockdown could reduce the proliferation and invasiveness of BC cells.

Furthermore, to verify the effect of apoptosis induced by knockdown of circRASSF2. The cell apoptosis was assessed by the expression of cleaved caspase-3 and cleaved PARP protein by western-blot, and detected the Annexin V-PE and 7AAD staining by Flow cytometry. It was revealed that there was a distinct drop in the number of apoptosis cells in the si-circRASSF2 group (Figure 3(d), p < 0.01); PCNA protein expression was significantly decreased, and the protein levels of cleaved-PARP and cleaved-caspase3 were significantly increased in the cells (Figure 2(e), p < 0.01), suggested that the decrease of circRASSF2 expression can promote apoptosis in BC cells.

### Knockdown of circRASSF2 inhibits migration and invasion of BC cells

**Figure 4.**
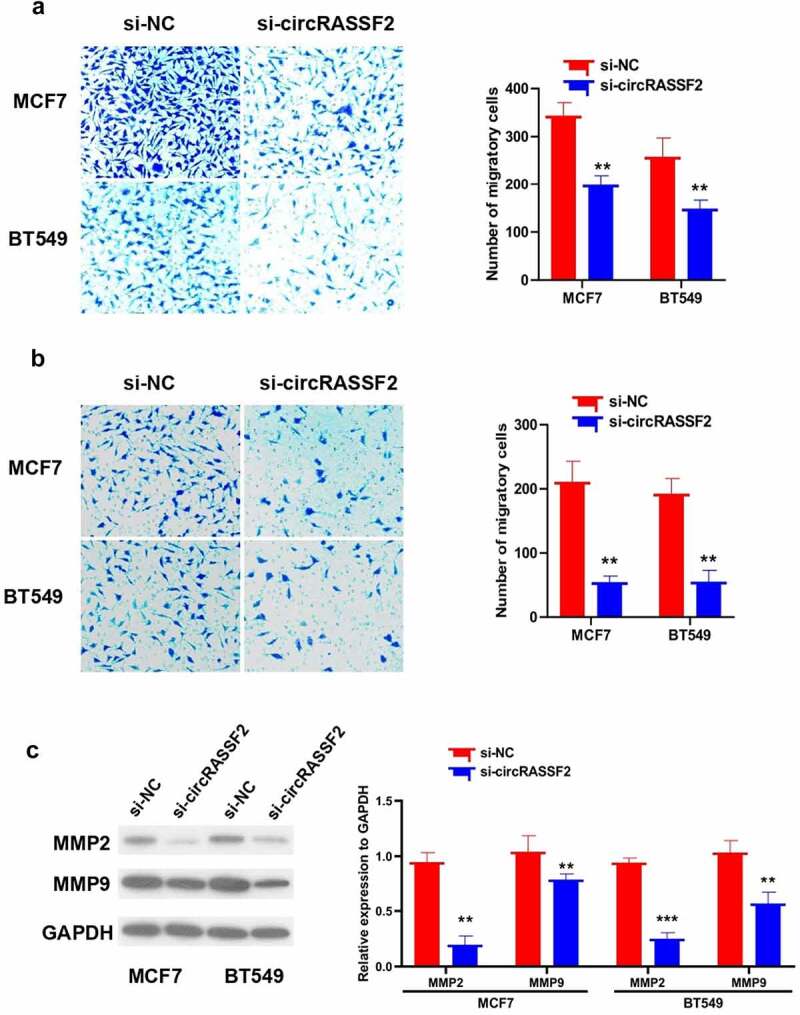
**Knockdown of circRASSF2 inhibits migration and invasion of BC cells**. (a) The migration ability of different groups of MCF7 and BT549 (si-NC and si-circRASSF2) was detected by Transwell (without matrix glue). (b) The invasive ability of different groups of MCF7 and BT549 (si-NC and si-circRASSF2) was detected by Transwell (with matrix glue). (c) Western blotting assay used to detect the protein levels of MMP2 and MMP9 in different groups of MCF7 and BT549 (si-NC and si-circRASSF2). *, P < 0.05; **, < 0.01; ***, P < 0.001

Assisted by Transwell migration and Matrigel invasion, cell migration and invasion assays were performed, showing that the number of cells crossing the membrane in si-circRASSF2 group was significantly less than that in si-NC group (Figure 4(a), p < 0.01). Therefore, it can be concluded that the up-regulated circRASSF2 could enhance the migration ability of BC cells. Based on transwell invasion assay, the number of cells crossing the membrane in si-circRASSF2 group was greatly smaller than that in si-NC group (Figure 4(b), p < 0.01), accounting for the promotional role of circRASSF2 group in the invasion of BC cells. In this study, to probe into what influence exerted by circRASSF2 on the migration and invasion ability of BC cells, the expression of invasion and migration-related proteins MMP2 and MMP9 was discerned by western blotting assay. As a consequence, circRASSF2 knockdown can reduce the protein expression levels of MMP2 and MMP9 in the cells (Figure 4(c), p < 0.01).

### CircRASSF2 up-regulates HOXA1 protein levels in BC cells via sponging miR-1205

Since circRNAs can act as miRNA sponges, this reduces the amount of miRNAs for binding to their target mRNAs. By searching circinteractome database, we found that the circRASSF2 sequence contains miRNA-1205 binding sites (Figure 4(a)), and to further confirm the relationship between circRASSF2 and miRNA-1205, we performed luciferase reporter gene assay in MCF7 and BT549 cells and the results showed that overexpression of miR-1205 was able to inhibit luciferase activity in cells compared to miR-NC, and the inhibiting effect would eliminate when the predicted circRASSF2 binding site were mutated (Figure 4(a)). To confirm the reliability of our results, qRT-PCR was put to use so as to detect the expression level of miR-1205 in MCF7 and BT549 cells after knockdown of circRASSF2, which demonstrated a remarkable rise in the expression of miR-1205 in cells in circRASSF2 kockdown cells (Figure 4(b), p < 0.05). It can therefore be inferred that circRASSF2 might participate in the development of BC via sponge miR-1205.

**Figure 5.**
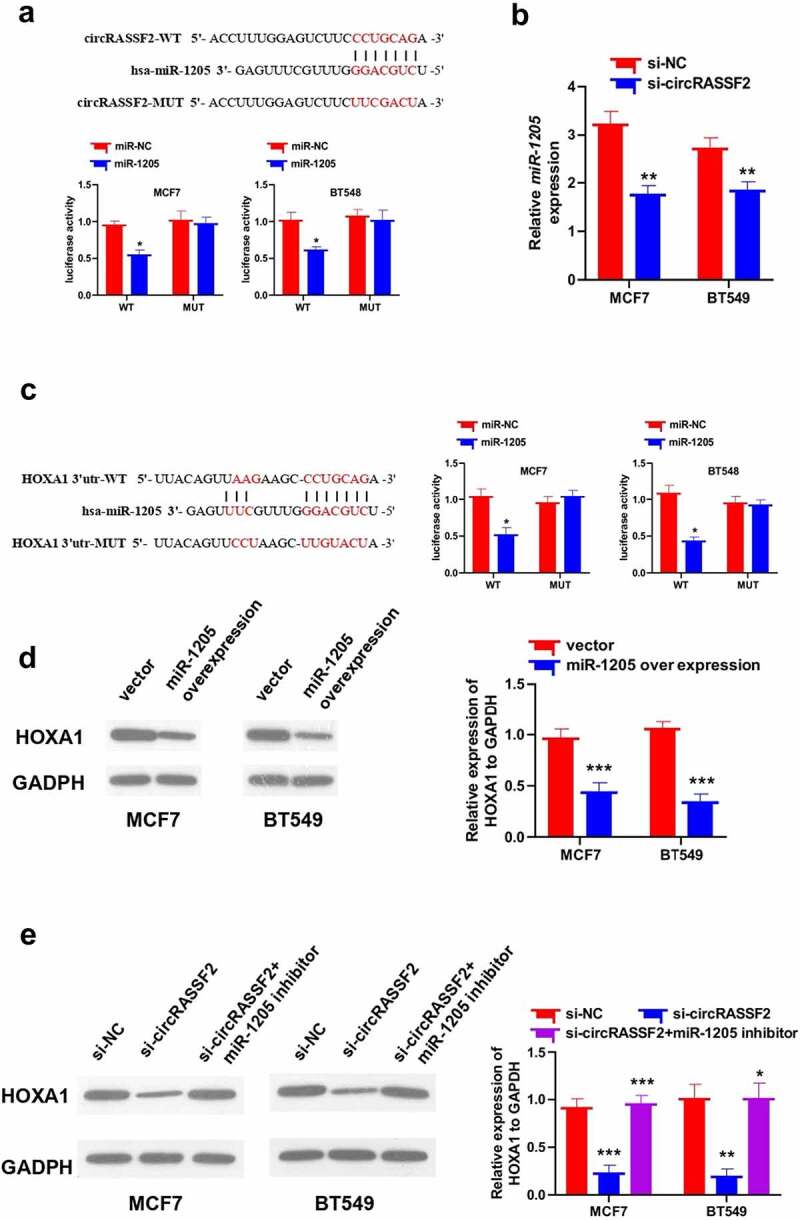
**CircRASSF2 up-regulates HOXA1 protein levels in BC cells via sponge miR-1205**. (a) Bioinformatics target prediction and dual luciferase reporter gene assay verified. (b) qRT-PCR method was used to detect the expression of miR-1205 in circRASSF2 knocking down MCF7 and BT549 cells. (c) Bioinformatics target prediction and dual luciferase reporter gene assay verified. (d) The HOXA1 protein expression levels in miR-1205 overexpression MCF7 and BT549 cells by Western blotting method. (e) The HOXA1 protein expression levels in MCF7 and BT549 (si-NC and si-circRASSF2, si-circRASSF2+ miR-1205 inhibitor). *, P < 0.05; **, < 0.01; ***, P < 0.001

By searching targetscan database, we found that the miR-1205 binding site on the 3ʹ-UTR of HOXA1, and to further confirm the relationship between HOXA1 and miR-1205, we performed luciferase reporter gene assay, and the results revealed that compared to miR-NC, overexpression of miR-1205 was able to refrain luciferase activity in cells, and that the efficacy of inhibition would be eliminated when the predicted HOXA1 3ʹ-UTR binding site were mutated (Figure 4(c)). To confirm the reliability of our results, we used Western blotting method to detect the HOXA1 expression in miR-1205 overexpression MCF7 and BT549 cells, revealing a great deduction in HOXA1 expression (Figure 4(d), p < 0.05); we also examined the HOXA1 expression levels in MCF7 and BT549 transfected with si-NC, si-circRASSF2 and si-circRASSF2+ miR-1205 inhibitor, the results showed that circRASSF2 knock down decrease the HOXA1 protein expression levels and the protein expression levels of HOXA1 were increased when co-transfected with miR-1205 inhibitor (Figure 5(e), p < 0.05).

## Disscussion

The molecular characteristics of BC and the diverse clinical manifestations of patients, the prognosis of patients is widely inconsistent, and the expected results cannot be achieved [2]. Therefore, figuring out the molecular mechanisms of BC progression so as to discover early diagnostic biomarkers and novel therapeutic targets for more effective treatment of BC is of importance.

As high-throughput sequencing technology continues to evolve, various expressions of circRNA have been discovered in multiple tumor tissues, including BC [23,24]. Normal BC tissues contain more circRNA than tumor tissues [25]. Besides, recent studies have confirmed that circRNA occupies a pivotal position in cancer progression by targeting different miRNAs, and may contribute to influence cell proliferation, invasion and metastasis [26–28]. Yin et al. [29] detected 41 differential expression circRNAs between normal and tumor tissues of the breast, including 19 with up-regulation and 22 with down-regulation, this result indicated that circRNA is related to BC and has potential for early diagnosis. In recent years, there are few reports about expression of circRASSF2 and human cancers. As stated by Wu et al. [8], acting as a ceRNA, circRASSF2 could activate the miR-1178/TLR4 signaling pathway to promote tumor progression in papillary thyroid cancer. Moreover, it is applicable to the regulation of miR-302B-3p/IGF-1 R axis to promote tumor progression in laryngeal squamous cell carcinoma [30]. In our study, it was found that the expression of circRASSF2 in BC tissues as well as BC cell lines was significantly up-regulated. We hypothesized that circRASSF2 is involved in the progression of BC. By further analyzing the effect of circRASSF2 on the pathological characteristics and prognosis of BC, the expression of circRASSF2 was demonstrated to have positive correlation with tumor size, differentiation, TNM stage, lymph node metastasis and distant metastasis. Moreover, the overall survival duration of BC patients in the group where circRASSF2 was highly expressed was far behind compared with that in the group with low expression of circRASSF2, thus revealing that the high level of circRASSF2 expression of is an influential risk factor in the progression of BC, and is a poor prognostic indicator of BC. ROC analysis with the expression of circRASSF2 in serum showed that high expression of circRASSF2 is promising to be a sensitive prognostic indicator of BC.

According to the functional study, the proliferation activity, clonogenic ability, migration and invasion ability were suppressed to a great extent in circRASSF2 knocking down cells. For the purpose of clarifying the possible mechanism of knockdown of circRASSF2 to promote the apoptosis of BC cells, the number of apoptosis cells was calculated by Flow cytometry, and Western blotting was implemented to detect the expression of PCNA, cleaved Caspase-3 and cleaved PARP in this study. Annexin-V is a sensitive indicator for detecting early apoptosis, and 7-AAD is a sensitive indicator for detecting apoptosis and apoptotic cells. We found an obvious decline of apoptosis in cells of MCF7 and BT549 with circRASSF2 knockdown, suggesting that circRASSF2 can regulate BC cell apoptosis. Proliferating cell nuclear antigen (PCNA) interacts with a variety of eukaryotic cell cycle-related proteins during the cell cycle process, and acts as an oncogene in a diversity of human cancers [31,32]. PCNA can participate in the synthesis and repair of DNA, and is the mutual aid protein of DNA polymerase δ [31]. Over proliferation is one of the main characteristics of tumors, studies have shown that PCNA has important significance in the proliferation of various tumors [32]. We found that PCNA expression was significantly reduced in circRASSF2 knock-down BC cells. Caspases belong to the family of cysteine proteases and play a key role in the apoptosis regulatory network. It is the common gathering point of multiple apoptotic pathways and is the final pathway that directly leads to the disintegration of apoptotic cells and the execution of apoptosis [33]. Caspase-3 is an important effector molecule for the execution of apoptosis, which is widely expressed in human normal and tumor tissues [34]. PARP is a multifunctional protein post-translational modified enzyme present in most eukaryotic cells, activated by identifying structurally given DNA fragments and associated with DNA damage and repair, and it is a key substrate of the caspase-3 [35]. Under the action of cells and various stimulating factors, the activated Caspase-3 can decompose PARP into different fragments to make it unable to perform normal functions, thereby initiating cell apoptosis [36]. In the end, both the cleaved Caspase-3 and the cleaved PARP expression were found to rise in circRASSF2 knocking down BC cells, suggesting activation of apoptotic pathways in these cells. Tumor cells must destroy extracellular matrix (ECM) during invasion and metastasis, and matrix metalloproteinases (MMPs) are essential to the progression of destroying the ECM [37]. The overexpression of MMP2 and MMP9 can be ascribed to the presence of a variety of malignant tumors [38,39]. In BC, MMP2 and MMP9 can be used as a resultful indicator of malignancy and prognosis, and may be a target for therapy [40]. We found that MMP2 and MMP9 expression were significantly inhibited in circRASSF2 knocking down BC cells, suggesting that circRASSF2 can regulate BC cells migration and invasion.

**Figure 6.**
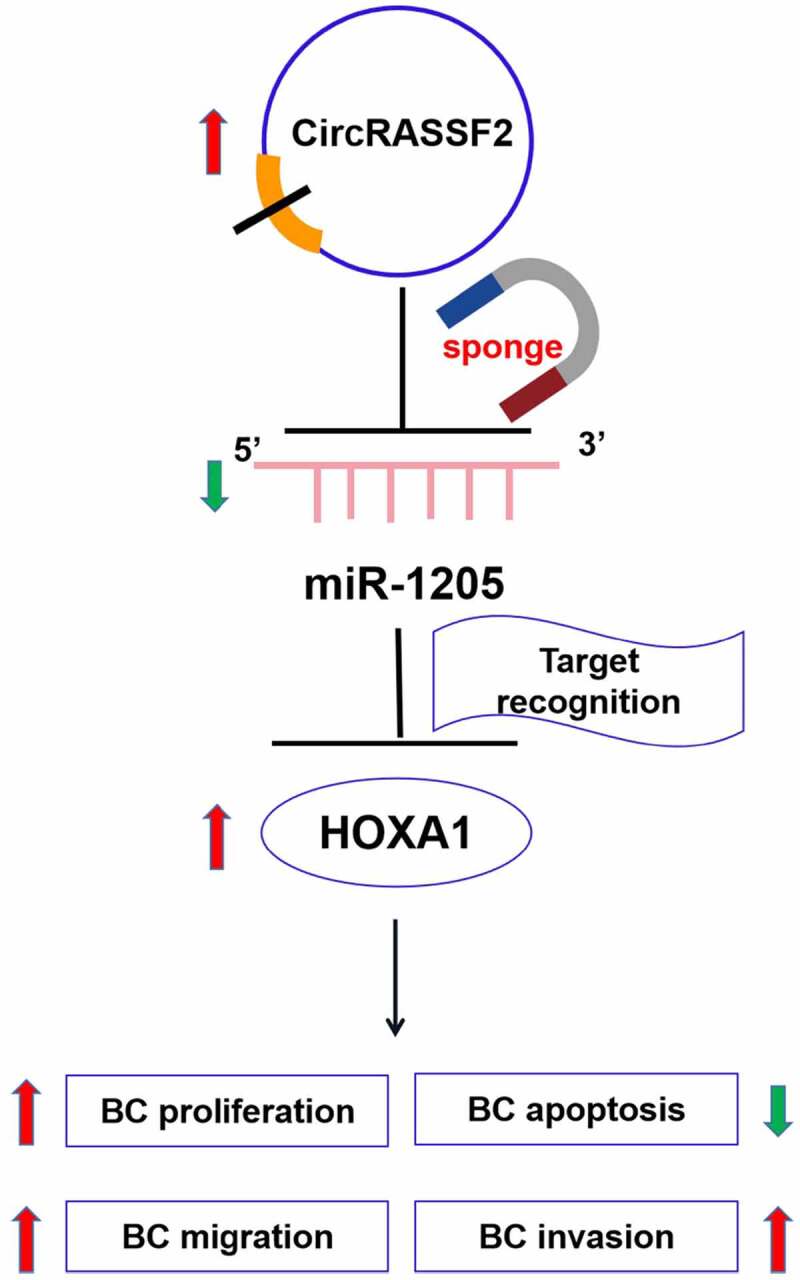
A schematic diagram showing the regulatory role of circRASSF2/miR-1205/HOXA1 axis in BC cells

We found that the circRASSF2 sequence contains miRNA-1205 binding sites, and confirmed by dual luciferase reporter assay. MiR-1205 is widely studied in tumors, in non-small cell lung cancer, miR-1205 exerts a tumor suppressor effect by cutting off the synergy between KRAS and MDM4/E2F1 [41]. In laryngeal squamous cell carcinoma, miR-1205 is down-regulated, which regulates tumor progression through interaction with E2F1 [42]. However, in BC, the expression level and function of miR-1205 are still unclear. By knocking down the expression of circRASSF2 in BC cells, it was found that the degree of expression of miR-1205 was negatively associated with that of circRASSF2 in BC cells. In order to further analyze the downstream target genes of miR-1205 in the regulation of miR-1205 by circRASSF2, targetscan was exploited to predict the target gene. The prediction results found that HOXA1 may be the target gene in the regulation network. We also applicated dual luciferase reported assay for further verification. The influence of circRASSF2 on miR-1205/HOXA1 regulation was confirmed by western blotting. It was discovered that the protein expression degree of HOXA1 was reduced in knocking down circRASSF2 cells. When miR-1205 inhibitor was co-transfected, the protein expression degree of HOXA1 in cells increased. In normal cells, HOXA1 is low or not expressed, but in the case of cell malignant transformation, HOXA1 immediately shows abnormally high expression [43]. HOXA1 is a key factor in tumor progression. Naturally expressed HOXA1 inhibitors have the ability to resist tumor progression and metastasis. The expression of HOXA1 in tumors is usually associated with the poor prognosis of cancer patients. HOXA1 may act as an oncogene to promote tumor progression [19,44]. In BC, studies have found that HOXA1 may be a necessary factor for the establishment of BC cell phenotype, HOXA1 gene may be one of the regulators regulating the differentiation of breast epithelial cells, and it possible that changes in HOXA1 expression are critical to the development of BC [45]. The increased expression of HOXA1 in breast epithelium is related to pathological proliferation [46]. The high expression of HOXA1 in human BC cells can promote tumor cell proliferation, mainly through the promotion of cell survival mediated by up-regulation of Bcl-2 transcription. At the same time, studies have also found that HOXA1 can inhibit the apoptotic response of BC cells to adriamycin, and the high expression of HOXA1 leads to canceration of human breast epithelial cells [47]. As shown in the schematic diagram (Figure 6), our results suggested that miR-1205 can regulate the occurrence and progression of BC by interacting with HOXA1 in BC cells, and this process is regulated by circRASSF2.

## Conclusion

To sum up, in this study, we believe that circRASSF2 is a vital cancer-promoting circRNA in the progression of BC. We found that circRASSF2 is highly expressed in BC and can act as a miR-1205 sponge to regulate HOXA1 expression to promote BC proliferation, migration and invasion. Through clinical specimens and cytology experiments, we believe that circRASSF2 could serve as a potential diagnostic biomarker and therapeutic target for BC.

## Limitation

In this study, the number of cases from specimen sources is relatively small. In future studies, we will need a larger number of specimens to determine the expression of circRASSF2 in the serum and tissues of BC patients. Since the structure of cirRNAs may have multiple miRNA binding sites, in the future research, we will further explore whether circRASSF2 can regulate other miRNAs in BC cells and whether it regulates the occurrence and development of BC through other regulatory pathways.

## Highlights

1. CircRASSF2 upregulation was correlated with the progression and poor prognosis in BC.
2. CircRASSF2 can be a good diagnosis marker for BC.
3. CircRASSF2 downregulation can inhibit BC cell proliferation and induce apoptosis.
4. CircRASSF2 downregulation can inhibit BC cell migration and invasion.
5. CircRASSF2 can up-regulate HOXA1 protein levels in BC cells via sponging miR-1205.
